# Trainee exposure to valve surgery in the UK: safety, decline and organisational inequality in a national cohort

**DOI:** 10.1136/openhrt-2026-004389

**Published:** 2026-10-01

**Authors:** Jeremy Chan, Maria Comanici, Pradeep Narayan, Tim Dong, Gianni D Angelini

**Affiliations:** 1 Bristol Heart Institute, Bristol, UK; 2 University of Bristol, Bristol, UK

**Keywords:** Inservice Training, Education, Medical, Heart Valve Diseases, Mitral Valve Insufficiency, Aortic Valve Stenosis

## Abstract

**Objective:**

To evaluate trends, early clinical outcomes and surgeon-variation and hospital-variation in trainees performing isolated aortic valve replacement (AVR) and mitral valve (MV) surgery, including replacement (MVR) and MV repair (MVr) in the UK.

**Methods:**

All adults undergoing elective or urgent isolated AVR or isolated MVr/MVR in the National Adult Cardiac Surgery Audit between 1996 and 2019 were included. Temporal trends and early clinical outcomes were compared between consultants and trainees as primary operators. Three-level multilevel logistic regression was used to quantify hospital-level and consultant-level variation in the proportion of cases performed by trainees.

**Results:**

Among 71 710 isolated AVR and 25 197 isolated MV surgery cases, 13 432 (18.7%) and 2472 (9.8%) were performed by trainees, respectively. MV surgery included 9893 MVR and 15 292 MVr, of which 1226 (12.4%) and 1246 (8.1%) were performed by trainees, respectively.

Propensity matching generated balanced cohorts of 13 432 AVR, 1223 MVR and 1243 MVr pairs. Trainee-led procedures were not associated with higher in-hospital morbidity or mortality.

Multilevel modelling demonstrated substantial variation in trainees being the primary operator, with 59.6%, 57.1% and 44.8% of variation at hospital-level and surgeon-level for AVR, MVr and MVR, respectively.

**Conclusions:**

In this national UK cohort, trainees performed approximately one in five isolated AVR operations and one in ten isolated MV operations. Trainee exposure was the lowest in MVr. Trainee-led procedures were not associated with an increase in in-hospital mortality or early morbidity. There was a marked hospital-level and consultant-level variation in trainees’ operating opportunities. These findings support continued structured exposure to isolated aortic and MV surgery, with particular attention to a specific MVr training pathway.

**WHAT IS ALREADY KNOWN ON THIS TOPIC**

- Selected cardiac surgical operations can be performed safely by trainees under supervision.

**WHAT THIS STUDY ADDS**

- Marked hospital-level and consultant-level variation in trainees’ operating opportunities in isolated aortic and MV surgery.

**HOW THIS STUDY MIGHT AFFECT RESEARCH, PRACTICE OR POLICY**

- Our study supports continued structured exposure to isolated aortic and MV surgery, with particular attention to a specific MVr training pathway.

## Introduction

 Operative competence in cardiac surgery requires supervised, graduated exposure across the spectrum of adult cardiac procedures. Coronary artery bypass grafting (CABG) has traditionally accounted for a substantial proportion of first-operator training opportunities, whereas valve surgery is often introduced later in the training in the UK.^1 2^ Contemporary training occurs within a more constrained environment, characterised by increasing patient complexity, evolving transcatheter therapies, reduced operative exposure related to working-time regulations, and consultant-level public reporting of outcomes.^2–11^

Aortic and mitral valve (MV) surgery presents distinct training challenges. Isolated aortic valve replacement (AVR) has a higher volume than MV surgery and is performed by most surgeons and all adult cardiac surgical units.^12^ In contrast, mitral surgery, particularly MV repair (MVr), has a lower volume, is more anatomically heterogeneous and is frequently concentrated among surgeons and centres with specialist repair expertise. Durable mitral repair depends not only on technical execution but also on valve analysis, exposure, repair planning and intraoperative judgement. Contemporary guidelines emphasise specialist valve expertise and volume-outcome literature reinforces the importance of institutional and surgeon experience, particularly for MVr.^3–6^ Although these developments may improve patient outcomes, they may also concentrate technically complex cases among experienced consultants and limit trainee first-operator exposure.

Previous studies suggest that selected cardiac surgical operations can be performed by supervised trainees without compromising early patient safety, including national UK analyses of advanced coronary surgery and single-centre or multicentre studies of AVR and MV surgery.^1 11 13–18^ However, national valve-specific evidence remains limited, particularly across long study periods. This study used a national audit cohort to describe trainee exposure in isolated AVR and MV surgery, to compare early outcomes between trainee and consultant first operators and to evaluate if there was any variation in training across hospitals and surgeons.

## Methods

### Data source and study design

This is a retrospective national cohort study using data from the National Adult Cardiac Surgery Audit (NACSA), a prospectively maintained registry of all adults undergoing cardiac surgical procedures in the UK. NACSA has been used extensively for national cardiac surgical outcome research.^12 19^

The isolated AVR and MV surgery cohorts included all adult patients undergoing elective or urgent isolated procedures between 1996 and April 2019. MV surgery was analysed overall and then separated into MV replacement (MVR) and repair. Patients undergoing CABG, additional valvular intervention, major aortic surgery, other cardiac procedures, previous cardiac surgery, emergency or salvage procedures or procedures with missing primary operator grade were excluded.

The primary descriptive outcome was the annual proportion of isolated AVR, all isolated MVR and MVr recorded with a trainee as the primary operator. Early clinical outcomes were compared between consultant and trainee first-operator cases before and after propensity score matching and after inverse probability treatment weighting (IPTW). Mortality was defined as death during the index admission.

### Statistical analysis

Continuous variables were summarised as median with IQR and compared using Wilcoxon rank-sum tests. Categorical variables were summarised as counts with percentages and compared using Pearson chi-squared tests. Propensity scores were estimated separately for the AVR, all-MV, MVR and MVr cohorts. A 1:1 nearest-neighbour propensity score matching approach was used to balance measured preoperative covariates between cases performed by consultants and trainees. Covariate balance was assessed using standardised mean differences, and IPTW was performed as a sensitivity analysis.^20–25^ Early outcomes were compared before and after adjustment. The prespecified matching covariates were age, sex, body mass index, left ventricular ejection fraction category, urgency, diabetes, smoking, pulmonary disease, peripheral vascular disease, preoperative atrial fibrillation, neurological dysfunction, previous myocardial infarction, creatinine-clearance category (or creatinine if that category was unavailable), Canadian Cardiovascular Society class, New York Heart Association class, previous percutaneous coronary intervention, poor mobility, interval myocardial infarction, preoperative ventilation, cardiogenic shock and preoperative inotrope use. European System for Cardiac Operative Risk Evaluation II (EuroSCORE II) was reported descriptively but was not entered as an additional matching covariate.

Three-level multilevel logistic regression models were fitted with operations at level 1, consultants at level 2 nested within hospitals, and hospitals at level 3. The dependent variable was whether a trainee was recorded as the first operator. Random intercepts were specified for consultants nested within hospitals and for hospitals because operations performed by the same consultant or within the same hospital are clustered and are not statistically independent. Model 1 was a null model. Model 2 adjusted for preoperative variables. Model 3 added socioeconomic status using the Index of Multiple Deprivation. Model four added consultant volume quartiles, and Model five added hospital volume quartiles. Variance components were used to calculate hospital and consultant-within-hospital variance partition coefficients (VPCs), which estimate the proportion of residual variation attributable to each provider level on the latent scale. The median OR (MOR) translates provider variance to the odds-ratio scale: it is the median difference in odds for two otherwise comparable operations in two randomly selected providers, comparing the provider with higher trainee exposure with the provider with lower exposure. An MOR of 1 indicates no provider-level heterogeneity.

R (V.4.2.3, R Foundation for Statistical Computing, Vienna, Austria) and R Studio (V.1.4.1103, RStudio, PBC) were used for statistical analysis. Graphs and tables were created using R (V.4.2.3, R Foundation for Statistical Computing, Vienna, Austria) and Microsoft Office 365 (V.16.0.14026, Microsoft Corporation, Washington, USA).

## Results

### Study population and temporal trends in trainee operating

A total of 71 710 isolated AVR operations and 25 197 isolated MV surgery operations were included in this study. Trainees performed 13 432 isolated AVR operations (18.7%) and 2472 all MV operations (9.8%). Among MV surgery, there were 9893 MVR, including 1226 trainees being the primary operator (12.4%), and 15 292 MVr, including 1246 cases performed by trainees (8.1%).

Trainee exposure differed between AVR and MV surgery. In isolated AVR, annual trainee operating remained relatively stable during the study period, ranging from 12.8% in 1998 to 22.1% in 2019. In all isolated MV surgery, trainee exposure was lower overall and declined from 18.0% in 1996 to 10.6% in 2019, with a nadir of 7.2% in 2015 (figure 1). Procedure-specific mitral trends showed higher overall trainee exposure in MVR than MVr (12.4% vs 8.1%). MVR exposure declined from 21.6% in 1996 to 13.8% in 2019, whereas MVr exposure was 9.0% in 2019 and peaked at 12.4% in 2011.

**Figure 1:**
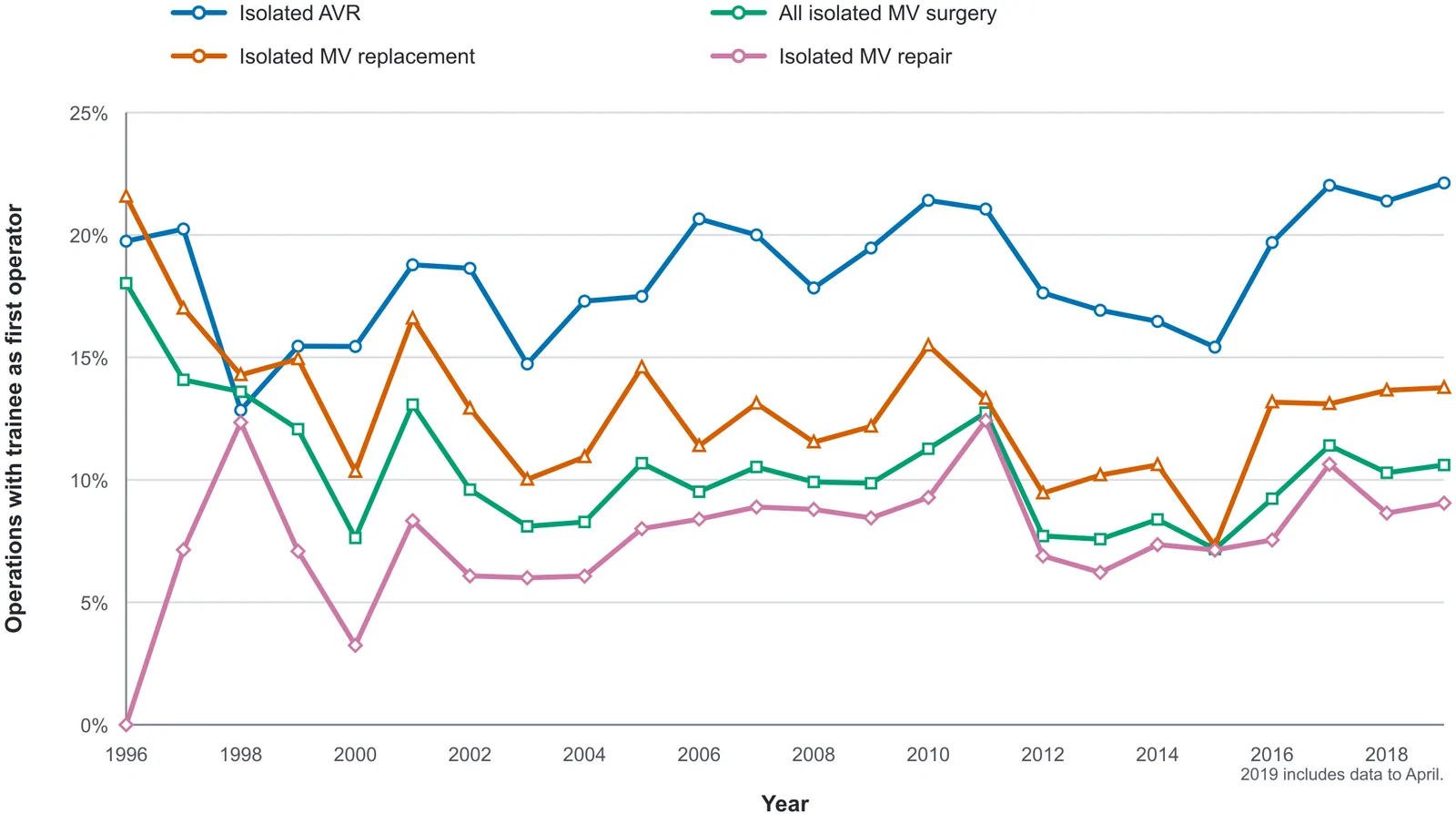
Annual proportion of isolated aortic valve replacement (AVR), all isolated mitral valve (MV) surgery, isolated MV replacement (MVR) and isolated MV repair (MVr) operations performed with a trainee as first operator, 1996–April 2019. The 2019 point includes procedures recorded to April.

### Multilevel modelling of training variation

Multilevel modelling demonstrated a substantial variation in trainee first-operator status at both hospital and consultant levels (table 1 and figure 2). In the final model, provider-level (surgeon+hospital) VPCs were 59.6% for isolated AVR, 57.1% for MVr and 44.8% for MVR. In isolated AVR, 34.4% of the variation was attributable to hospitals and 25.2% to consultants within hospitals. In MVr, consultant variation accounted for a larger share than hospital variation (33.1% vs 24.0%). In MVR, hospital and consultant components were similar (23.3% and 21.5%, respectively).

**Figure 2:**
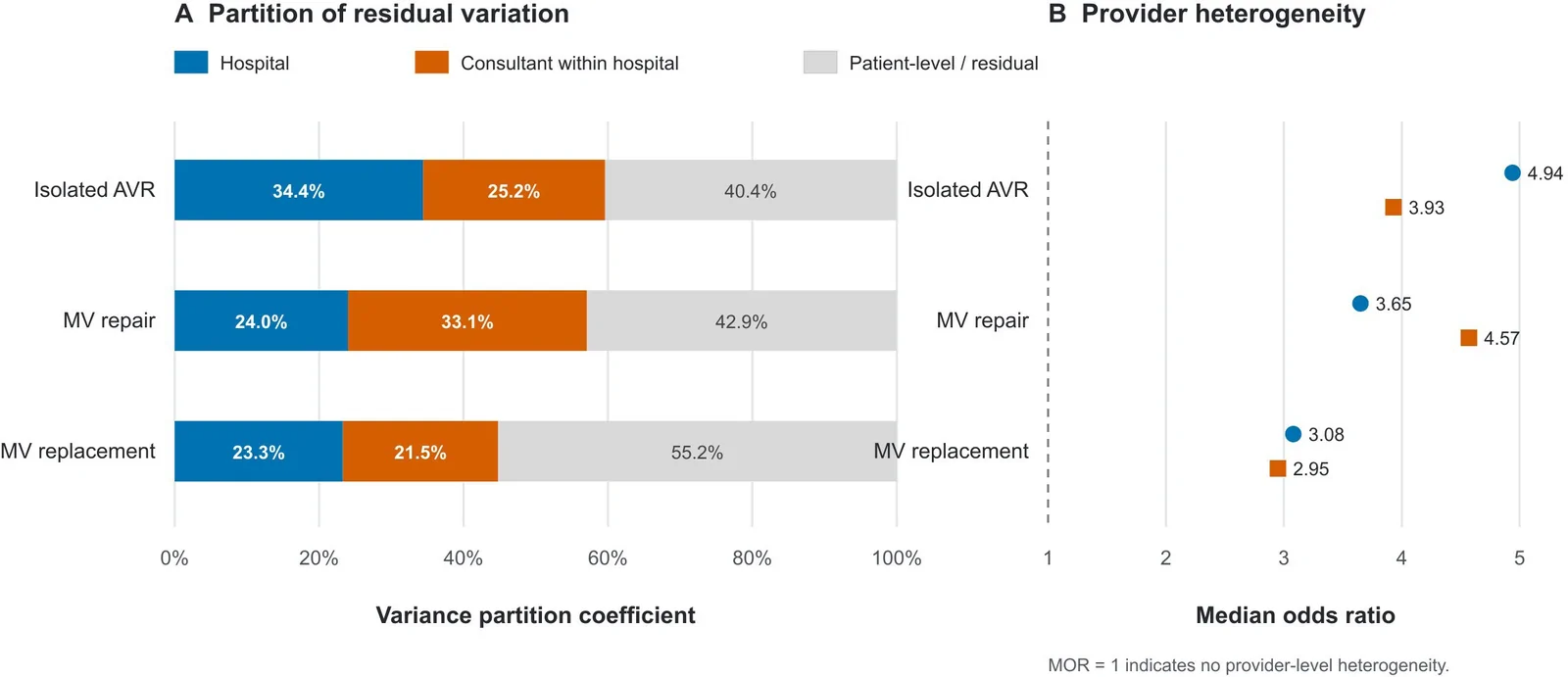
Provider-level variation in trainee first-operator status from the final three-level multilevel logistic regression models. (A) Hospital, consultant-within-hospital and patient-level/residual variance partition coefficients for isolated AVR, MV repair and MV replacement. (B) The corresponding median ORs (MORs); MOR=1 indicates no provider-level heterogeneity. AVR, aortic valve replacement; MV, mitral valve.

**Table 1:** Multilevel logistic regression variance components for trainee first-operator status in isolated AVR, MV repair and MV replacement

Cohort	Operations; hospitals; consultant-hospital groups	Null provider VPC, %	Final hospital VPC; MOR	Final consultant VPC; MOR	Final provider VPC, %	Key final-model volume finding
Isolated AVR	71 710; 44; 1101	60.0	34.4%; MOR 4.94	25.2%; MOR 3.93	59.6	Consultant volume Q4 versus Q1: OR 1.74 (95% CI 1.17 to 2.58), p=0.006; hospital volume not significant.
Isolated MV repair	15 292; 44; 634	60.7	24.0%; MOR 3.65	33.1%; MOR 4.57	57.1	Hospital volume Q2 versus Q1: OR 4.64 (95% CI 1.44 to 14.96), p=0.010; consultant volume not significant.
Isolated MV replacement	9893; 43; 755	45.9	23.3%; MOR 3.08	21.5%; MOR 2.95	44.8	No significant consultant-volume or hospital-volume association in the final model.

Final model: preoperative variables+Index of Multiple Deprivation+consultant vol quartile+hospital vol quartile.

Provider VPC combines hospital and consultant-within-hospital variance on the latent scale.

AVR, aortic valve replacement; MOR, median OR; MV, mitral valve; VPC, variance partition coefficient.

The final-model hospital MOR was 4.94 for AVR, 3.65 for MVr, and 3.08 for MVR. The corresponding consultant MORs were 3.93, 4.57 and 2.95. Consultant volume was independently associated with trainee first-operator status in AVR, with the highest consultant volume quartile having higher odds than the lowest quartile (OR 1.74, 95% CI 1.17 to 2.58; p=0.006). In MVr, hospital volume showed the strongest association, with higher odds in the second hospital volume quartile compared with the lowest quartile (OR 4.64, 95% CI 1.44 to 14.96; p=0.010). No statistically significant consultant- or hospital-volume association was observed in the final MVR model.

### Early outcomes after propensity score matching

Propensity score matching generated balanced cohorts of 26 864 AVR patients, 4944 all MV patients, 2446 MVR replacement patients, and 2486 MVr patients (online supplemental tables 1 and 2). In the matched AVR cohort, cases performed by trainees had longer cardiopulmonary bypass time (90 vs 84 min, p<0.001) and aortic cross-clamp time (69 vs 62 min, p<0.001). There were no significant differences in return to theatre (6.0% vs 6.0%, p=0.78), return to theatre for bleeding or tamponade (4.4% vs 4.4%, p=0.83), postoperative cerebrovascular events (1.5% vs 1.3%, p=0.34), deep sternal wound infection (0.5% vs 0.4%, p=0.63), or total length of stay (9 vs 9 days, p=0.089). Mortality (1.9% vs 1.2%, p<0.001) and postoperative dialysis (2.4% vs 1.8%, p=0.001) were lower in trainee first-operator cases (table 2).

**Table 2:** Isolated AVR operative and early postoperative outcomes before PSM, after PSM and after IPTW

Outcome	Before PSM			After PSM			After IPTW		
Outcome	Consultant (n=58 278)	Trainee (n=13 432)	P value	Consultant (n=13 432)	Trainee (n=13 432)	P value	Consultant (weight=58 278)	Trainee (weight=13 274)	P value
CPB, min	84 (67, 105)	90 (75, 109)	<0.001	84 (67, 105)	90 (75, 109)	<0.001	84 (67, 105)	90 (75, 109)	<0.001
Cross-clamp, min	62 (49, 79)	69 (56, 84)	<0.001	62 (50, 79)	69 (56, 84)	<0.001	62 (49, 79)	69 (56, 84)	<0.001
Total length of stay, days	9 (7, 13)	9 (7, 13)	<0.001	9 (7, 13)	9 (7, 13)	0.089	9 (7, 13)	9 (7, 13)	<0.001
Mortality	1141/57 999 (2.0%)	157/13333 (1.2%)	<0.001	250/13 386 (1.9%)	157/13 333 (1.2%)	<0.001	1115/57 998 (1.9%)	166/13 178 (1.3%)	<0.001
Return to theatre	3163/52 769 (6.0%)	722/11 946 (6.0%)	0.84	726/12 187 (6.0%)	722/11 946 (6.0%)	0.78	3155/52 784 (6.0%)	713/11 795 (6.0%)	0.8
Bleeding/tamponade return to theatre	2560/58 278 (4.4%)	593/13 432 (4.4%)	0.91	586/13 432 (4.4%)	593/13 432 (4.4%)	0.83	2556/58 278 (4.4%)	582/13 274 (4.4%)	>0.9
Postoperative CVA/TIA	784/51 934 (1.5%)	177/11 777 (1.5%)	0.50	161/11 932 (1.3%)	177/11 777 (1.5%)	0.34	780/51 924 (1.5%)	181/11 648 (1.6%)	0.7
Postoperative dialysis	1208/52 361 (2.3%)	203/11 478 (1.8%)	<0.001	287/12 060 (2.4%)	203/11 478 (1.8%)	0.001	1187/52 362 (2.3%)	210/11 343 (1.9%)	0.008
Deep sternal wound infection	120/19 807 (0.6%)	21/4 062 (0.5%)	0.5	21/4704 (0.4%)	21/4062 (0.5%)	0.63	120/19 866 (0.6%)	22/3994 (0.5%)	0.6
DSWI treatment	95/19 782 (0.5%)	16/4 057 (0.4%)	0.5	16/4,699 (0.3%)	16/4,057 (0.4%)	0.52	95/19 841 (0.5%)	16/3988 (0.4%)	0.6

Continuous variables are median (Q1, Q3); categorical variables are n/N (%).

AVR, aortic valve replacement; Consultant, consultant first operator; CPB, cardiopulmonary bypass; CVA/TIA, cerebrovascular accident/transient ischaemic attack; DSWI, deep sternal wound infection; IPTW, inverse probability of treatment weighting; PSM, propensity score matching; trainee, trainee first operator; weight, IPTW weighted total.

In the matched all MV cohort, cardiopulmonary bypass time was similar between groups (97 vs 95 min, p=0.21), whereas aortic cross-clamp time was longer in trainee first-operator cases (74 vs 70 min, p=0.004). Total length of stay did not differ (9 vs 9 days, p=0.82). In-hospital mortality (2.2% vs 1.2%, p=0.004), return to theatre (6.7% vs 5.2%, p=0.034), and postoperative dialysis (2.6% vs 1.5%, p=0.007) were higher in cases performed by consultants (table 3).

**Table 3:** All isolated MV surgery operative and early postoperative outcomes before PSM, after PSM and after IPTW

Outcome	Before PSM			After PSM			After IPTW		
Outcome	Consultant (n=22 725)	Trainee (n=2472)	P value	Consultant (n=2472)	Trainee (n=2472)	P value	Consultant (weight=22 725)	Trainee (weight=2418)	P value
CPB, min	96 (77, 124)	97 (81, 117)	0.60	95 (77, 123)	97 (81, 117)	0.21	96 (77, 124)	97 (81, 118)	0.2
Cross-clamp, min	71 (56, 93)	74 (60, 89)	0.004	70 (56, 91)	74 (60, 89)	0.004	71 (56, 93)	74 (61, 90)	<0.001
Total length of stay, days	9 (7, 14)	9 (7, 14)	0.002	9 (7, 14)	9 (7, 14)	0.82	9 (7, 14)	9 (7, 14)	0.4
Mortality	470/22 569 (2.1%)	29/2445 (1.2%)	0.003	55/2454 (2.2%)	29/2445 (1.2%)	0.004	474/22 570 (2.1%)	26/2393 (1.1%)	<0.001
Return to theatre	1194/20 590 (5.8%)	112/2154 (5.2%)	0.3	150/2233 (6.7%)	112/2154 (5.2%)	0.034	1197/20 585 (5.8%)	108/2109 (5.1%)	0.2
Bleeding/tamponade return to theatre	879/22 725 (3.9%)	88/2472 (3.6%)	0.4	108/2472 (4.4%)	88/2472 (3.6%)	0.14	881/22 725 (3.9%)	83/2418 (3.4%)	0.3
Postoperative CVA/TIA	299/19 778 (1.5%)	40/2231 (1.8%)	0.3	30/2142 (1.4%)	40/2231 (1.8%)	0.39	302/19 783 (1.5%)	36/2171 (1.7%)	0.6
Postoperative dialysis	448/19 947 (2.2%)	30/2068 (1.5%)	0.018	57/2169 (2.6%)	30/2068 (1.5%)	0.007	451/19 947 (2.3%)	27/2020 (1.3%)	0.005
Deep sternal wound infection	32/7076 (0.5%)	1/503 (0.2%)	0.7	4/714 (0.6%)	1/503 (0.2%)	0.65	32/7041 (0.5%)	1/514 (0.3%)	0.6
DSWI treatment	26/7070 (0.4%)	1/503 (0.2%)	>0.9	3/713 (0.4%)	1/503 (0.2%)	0.76	26/7035 (0.4%)	1/514 (0.3%)	0.7

Continuous variables are median (Q1, Q3); categorical variables are n/N (%).

Consultant, consultant first operator; CPB, cardiopulmonary bypass; CVA/TIA, cerebrovascular accident/transient ischaemic attack; DSWI, deep sternal wound infection; IPTW, inverse probability of treatment weighting; MV, mitral valve; PSM, propensity score matching; trainee, trainee first operator; weight, IPTW weighted total.

Procedure-specific analysis showed that matched MVR trainee cases had longer bypass time (96 min vs 91 min, p<0.001) and cross-clamp time (73 min vs 66.5 min, p<0.001), while mortality, return to theatre and dialysis were lower in trainee first-operator cases. In matched MVr, bypass time, cross-clamp time, and length of stay were similar between groups; mortality was lower in trainee first-operator cases (0.7% vs 1.6%, p=0.040), with no significant difference in return to theatre, dialysis, cerebrovascular events, or deep sternal wound infection. IPTW analyses were consistent with no excess early adverse outcomes in trainee first-operator cases, although operative time differences persisted in MVR (online supplemental tables 3 and 4).

## Discussion

This national cohort study demonstrates that supervised trainee first-operator operating in isolated AVR and isolated MV surgery was not associated with worse early clinical outcomes after adjustment for measured case mix. Trainee exposure was substantially higher for AVR than for MV surgery. AVR remained a relatively preserved training operation across the study period, whereas trainee first-operator exposure in MV surgery was low and declined over time. Procedure-specific mitral analyses showed important heterogeneity, with lower trainee exposure in MVr than MVR.

Trainee first-operator cases had longer bypass and cross-clamp times in AVR and MVR, a pattern consistent with previous valve and broader cardiac surgical training literature.^11 13–18^ Importantly, longer operative times in this analysis were not accompanied by increased in-hospital mortality and morbidities. In MVr, matched operative times were similar between consultants and trainees.

The higher level of trainee exposure in AVR is consistent with existing literature. A prior single-centre UK study reported that trainee first-operator status in isolated surgical AVR was not associated with worse early or late outcomes, despite longer operative times and changing patient risk profiles.^13^ This aligns with broader reports showing that selected cardiac operations, including valve surgery, can be performed safely by supervised trainees when cases are appropriately selected, and consultant support is available.^11 17 18^ The present national findings are concordant with those observations and extend them from a single-centre experience to a national cohort, supporting supervised AVR is feasible and safe.

Mitral surgery presents a different training environment. The lower trainee exposure observed in the overall mitral cohort, and particularly in MVr, likely reflects lower volume, greater technical heterogeneity, decision-making during repair and the importance of durable valve competence in addition to early postoperative safety. MVr outcomes are closely linked to surgical expertise and institutional experience, and contemporary guidelines recommend management in settings with appropriate valve expertise.^3–6^ The present findings suggest that supervised trainee exposure can be delivered without an early safety penalty, but the scale of the disparity deserves emphasis. Trainees performed 8.1% of MVr cases compared with 12.4% of MVR cases. Repair is the more technically demanding procedure and the one contemporary guideline preferentially recommends. Incidentally, this is precisely where trainee exposure is lowest. This gap is not incidental; it reflects the compounded effect of lower overall mitral volumes, greater technical selectivity and repair-specific risk aversion, and thus represents the most fragile point in the current training pathway.

The mitral findings are also consistent with the available trainee literature, but they highlight a more fragile training environment. Single-centre and multicentre studies of mitral surgery have reported comparable early and mid-term outcomes between trainees and consultants after adjustment, although trainee cases were often selected and comprised a smaller proportion of total mitral volume.^14–16^

Transcatheter valve therapies are likely to further influence this training balance. The expansion of transcatheter aortic valve implantation (TAVI) into lower-risk populations and the increasing availability of mitral transcatheter edge-to-edge repair have changed valve pathways and may alter the case mix remaining for surgical training.^26–32^ Simpler or higher-risk-but-transcatheter-suitable cases may be diverted from conventional surgery, whereas surgical programmes may retain a greater proportion of anatomically complex cases, redo operations, endocarditis, failed transcatheter interventions or cases requiring concomitant procedures. Surgical explantation after TAVI and mitral surgery after failed transcatheter edge-to-edge repair are associated with substantial operative risk and technical complexity, with low rates of successful repair after failed Transcatheter Edge-to-Edge Repair (TEER) in several reports.^30 33–35^ These cases are unlikely to be appropriate opportunities for trainees. Consequently, programmes may need to protect carefully selected conventional valve cases for training while ensuring that consultants maintain expertise in complex surgery and failed-transcatheter rescue operations. Because post-April 2019 operator-grade data were not available in the approved analytic extract, the present analysis cannot quantify the combined effect of the pandemic and subsequent transcatheter expansion on trainee exposure. A subsequent national analysis from January 2018 to March 2022 documented pandemic-related disruption to UK cardiac surgical services, including longer waiting times and changes in operative urgency and mortality.^36^ Contemporary follow-up with operator-grade data is therefore needed.

These findings have practical implications for training programme design. The current UK and Ireland cardiothoracic curriculum is outcome-based and requires major first-operator experience, while recent analyses suggest that CABG and AVR constitute most of the cardiac-themed operative exposure, with AVR representing a smaller but important component of logged major cases.^2 8^ Analysis of the study period reveals a concerning directional signal. The trainee first-operator exposure in MV surgery declined over the 1996–2019 window, while AVR exposure, though more preserved, did not demonstrably increase despite rising overall caseload. It is important to note that this decline began well before transcatheter therapies were introduced and perhaps reflects the effect of working-time restrictions, increasing complexity of cases, and cautious case selection over two decades. Due to these changes, the trainee exposure has already been shrinking for twenty years, and the decline perhaps accelerating with the arrival of TAVI and transcatheter mitral therapies.

Training requirements should distinguish the core competence expected of all cardiothoracic trainees from advanced subspecialist competence in complex MVr. All trainees require sufficient exposure to assess valve pathology, understand repair strategy, perform essential operative steps under supervision and manage common intraoperative problems; however, it may be neither feasible nor necessary for every trainee to complete training as an independent operator for the full spectrum of complex mitral repair. It is also worth mentioning that MVR is an index operation in the updated cardiothoracic surgery curriculum that all trainees are required to achieve by the end of the training. Trainees intending a mitral subspecialty would require additional concentrated experience in high-volume valve centres, potentially through regional rotations, post-certification fellowships and structured simulation. The aim should therefore be equitable access to a defined core mitral curriculum, with a separate advanced pathway for future mitral specialists. Nationally audited logbook data and proficiency-based assessments could identify programmes where core exposure is inadequate and support timely remediation.

The multilevel findings add an organisational dimension to these observations. Substantial residual provider-level variation persisted after adjustment for measured patient factors, socioeconomic status, consultant volume and hospital volume, indicating that trainee access to valve operating is shaped by where and with whom trainees operate as well as by case mix. For AVR, 59.6% of latent-scale residual variation was attributable jointly to providers: 34.4% between hospitals and 25.2% between consultants within hospitals. The hospital MOR of 4.94 means that, for two otherwise comparable AVR operations at two randomly selected hospitals, the median difference in the odds of a trainee being first operator is 4.94-fold when the hospital with higher trainee exposure is compared with the hospital with lower exposure; it does not compare the single highest- and lowest-exposure hospitals. The pattern differed by procedure: AVR variation was shared between hospitals and consultants, MVr showed particularly prominent consultant variation, and MV replacement showed a lower but still clinically important provider-level component. These findings support training strategies at both institutional and consultant-team levels rather than reliance on national case-volume targets alone.

## Limitations

This study has limitations inherent to retrospective observational analyses of audit data. First-operator grade does not capture the exact proportion of the operation performed by the trainee, trainee seniority, consultant scrubbed status, or intraoperative takeover. The analysis therefore evaluates recorded first-operator status rather than unsupervised trainee autonomy.

Second, propensity score methods can balance measured baseline variables but cannot eliminate unmeasured confounding. Valve anatomy, repair complexity, annular calcification, frailty, intraoperative judgement, surgeon and centre volume and granular echocardiographic features were not available in the supplied results. The lower mortality and dialysis rates observed in trainee cases are particularly susceptible to residual selection bias, which is especially relevant when interpreting the procedure-specific MVR and MVr analyses.

Third, the available outcomes were limited to early postoperative endpoints. Long-term survival, valve durability, recurrent mitral regurgitation, prosthesis-related complications, reoperation, quality of life and patient-reported outcomes were not available. These endpoints are particularly important for MVr, for which early safety does not fully capture technical quality. Finally, the study period ended in April 2019 and therefore does not capture the effects of the COVID-19 pandemic or the most recent expansion of transcatheter valve therapies. The approved analytic extract did not include post-April 2019 operator-grade data, so the present trainee first-operator analysis could not be extended through the pandemic. Accordingly, these pre-pandemic exposure estimates should not be assumed to represent current practice; updated analysis is required to evaluate both pandemic recovery and the continuing expansion of transcatheter therapies.

## Conclusion

In this national UK cohort study, isolated AVR and isolated MV surgery performed by trainees was not associated with worse early clinical outcomes after adjustment. Trainees performed approximately one-fifth of isolated AVR operations and one-tenth of isolated MV operations. Trainee exposure was lower in MVr than in MVR, but there was no signal of worse early safety outcomes in either subgroup.

Multilevel modelling demonstrated marked hospital- and consultant-level variation in trainee first-operator status, reinforcing that training exposure is an organisational outcome as well as a function of case selection. These findings support continued supervised trainee exposure to isolated aortic and MV surgery, with particular attention to structured MVr pathways in the era of advancing transcatheter valve practice.

## Supplementary material

10.1136/openhrt-2026-004389 online supplemental file 1

## Data Availability

No data are available.
