# Supplementary material for: Trainee exposure to valve surgery in the UK: safety, decline and organisational inequality in a national cohort
Source: Open Heart. 2026 Oct 1;13(2):e004389. doi: 10.1136/openhrt-2026-004389 (PMC13630039; doi:10.1136/openhrt-2026-004389)
Supplement: Supplementary file 1 [file openhrt-13-2-s001.docx]

**Supplementary Table 1. Isolated AVR preoperative characteristics before and after propensity score matching.**

| **Characteristic** | **Statistic/level** | **Before PSM** | | | **After PSM** | | | |
| --- | --- | --- | --- | --- | --- | --- | --- | --- |
| **Characteristic** | **Statistic/level** | **Consultant (n=58,278)** | **Trainee (n=13,432)** | **p value** | **Consultant (n=13,432)** | **Trainee (n=13,432)** | **SMD** | **p value** |
| Age, years | Median (Q1, Q3) | 70.30 (61.30, 77.20) | 70.10 (61.90, 76.60) | 0.25 | 70.40 (61.50, 77.10) | 70.10 (61.90, 76.60) | 0.005 | 0.092 |
| Gender | 0 | 33,871/58,278 (58%) | 8,065/13,432 (60%) | <0.001 | 8,020/13,432 (60%) | 8,065/13,432 (60%) | 0.007 | 0.73 |
| Gender | 1 | 24,397/58,278 (42%) | 5,364/13,432 (40%) |  | 5,410/13,432 (40%) | 5,364/13,432 (40%) |  |  |
| Gender | Missing | 10/58,278 (<0.1%) | 3/13,432 (<0.1%) |  | 2/13,432 (<0.1%) | 3/13,432 (<0.1%) |  |  |
| BMI | Median (Q1, Q3) | 27.68 (24.68, 31.14) | 27.68 (24.88, 31.12) | 0.027 | 27.68 (24.80, 31.03) | 27.68 (24.88, 31.12) | 0.012 | 0.093 |
| LVEF category | 0 | 38/58,278 (<0.1%) | 10/13,432 (<0.1%) | <0.001 | 10/13,432 (<0.1%) | 10/13,432 (<0.1%) | 0.008 | 0.89 |
| LVEF category | 1 | 11,740/58,278 (20%) | 2,205/13,432 (16%) |  | 2,178/13,432 (16%) | 2,205/13,432 (16%) |  |  |
| LVEF category | 2 | 805/58,278 (1.4%) | 182/13,432 (1.4%) |  | 169/13,432 (1.3%) | 182/13,432 (1.4%) |  |  |
| LVEF category | 3 | 44,241/58,278 (76%) | 10,760/13,432 (80%) |  | 10,784/13,432 (80%) | 10,760/13,432 (80%) |  |  |
| LVEF category | Missing | 1,454/58,278 (2.5%) | 275/13,432 (2.0%) |  | 291/13,432 (2.2%) | 275/13,432 (2.0%) |  |  |
| Urgency | 1 | 47,229/58,278 (81%) | 11,237/13,432 (84%) | <0.001 | 11,314/13,432 (84%) | 11,237/13,432 (84%) | 0.016 | 0.20 |
| Urgency | 2 | 11,049/58,278 (19%) | 2,195/13,432 (16%) |  | 2,118/13,432 (16%) | 2,195/13,432 (16%) |  |  |
| Diabetes | 0 | 50,108/58,278 (86%) | 11,428/13,432 (85%) | 0.001 | 11,454/13,432 (85%) | 11,428/13,432 (85%) | 0.008 | 0.89 |
| Diabetes | 1 | 1,741/58,278 (3.0%) | 408/13,432 (3.0%) |  | 419/13,432 (3.1%) | 408/13,432 (3.0%) |  |  |
| Diabetes | 2 | 4,825/58,278 (8.3%) | 1,249/13,432 (9.3%) |  | 1,217/13,432 (9.1%) | 1,249/13,432 (9.3%) |  |  |
| Diabetes | 3 | 1,604/58,278 (2.8%) | 347/13,432 (2.6%) |  | 342/13,432 (2.5%) | 347/13,432 (2.6%) |  |  |
| Smoking | 0 | 27,919/58,278 (48%) | 6,247/13,432 (47%) | <0.001 | 6,386/13,432 (48%) | 6,247/13,432 (47%) | 0.021 | 0.23 |
| Smoking | 1 | 25,680/58,278 (44%) | 6,195/13,432 (46%) |  | 6,068/13,432 (45%) | 6,195/13,432 (46%) |  |  |
| Smoking | 2 | 4,679/58,278 (8.0%) | 990/13,432 (7.4%) |  | 978/13,432 (7.3%) | 990/13,432 (7.4%) |  |  |
| Pulmonary disease | 0 | 50,340/58,278 (86%) | 11,762/13,432 (88%) | <0.001 | 11,766/13,432 (88%) | 11,762/13,432 (88%) | 0.001 | 0.94 |
| Pulmonary disease | 1 | 7,938/58,278 (14%) | 1,670/13,432 (12%) |  | 1,666/13,432 (12%) | 1,670/13,432 (12%) |  |  |
| Peripheral vascular disease | 0 | 54,520/58,278 (94%) | 12,770/13,432 (95%) | <0.001 | 12,750/13,432 (95%) | 12,770/13,432 (95%) | 0.007 | 0.58 |
| Peripheral vascular disease | 1 | 3,758/58,278 (6.4%) | 662/13,432 (4.9%) |  | 682/13,432 (5.1%) | 662/13,432 (4.9%) |  |  |
| Preoperative AF | 0 | 52,443/58,278 (90%) | 12,283/13,432 (91%) | <0.001 | 12,301/13,432 (92%) | 12,283/13,432 (91%) | 0.005 | 0.69 |
| Preoperative AF | 1 | 5,835/58,278 (10%) | 1,149/13,432 (8.6%) |  | 1,131/13,432 (8.4%) | 1,149/13,432 (8.6%) |  |  |
| Neurological dysfunction | 0 | 56,776/58,278 (97%) | 13,099/13,432 (98%) | 0.52 | 13,077/13,432 (97%) | 13,099/13,432 (98%) | 0.011 | 0.40 |
| Neurological dysfunction | 1 | 1,502/58,278 (2.6%) | 333/13,432 (2.5%) |  | 355/13,432 (2.6%) | 333/13,432 (2.5%) |  |  |
| Previous MI | 0 | 55,313/58,278 (95%) | 12,859/13,432 (96%) | <0.001 | 12,848/13,432 (96%) | 12,859/13,432 (96%) | 0.007 | 0.83 |
| Previous MI | 1 | 2,687/58,278 (4.6%) | 523/13,432 (3.9%) |  | 528/13,432 (3.9%) | 523/13,432 (3.9%) |  |  |
| Previous MI | 2 | 278/58,278 (0.5%) | 50/13,432 (0.4%) |  | 56/13,432 (0.4%) | 50/13,432 (0.4%) |  |  |
| Creatinine clearance category | 0 | 3,228/58,278 (5.5%) | 668/13,432 (5.0%) | 0.002 | 698/13,432 (5.2%) | 668/13,432 (5.0%) | 0.013 | 0.61 |
| Creatinine clearance category | 1 | 11,643/58,278 (20%) | 2,809/13,432 (21%) |  | 2,738/13,432 (20%) | 2,809/13,432 (21%) |  |  |
| Creatinine clearance category | 2 | 43,157/58,278 (74%) | 9,914/13,432 (74%) |  | 9,951/13,432 (74%) | 9,914/13,432 (74%) |  |  |
| Creatinine clearance category | 3 | 250/58,278 (0.4%) | 41/13,432 (0.3%) |  | 45/13,432 (0.3%) | 41/13,432 (0.3%) |  |  |
| CCS class | 0 | 34,520/58,278 (59%) | 7,805/13,432 (58%) | <0.001 | 7,928/13,432 (59%) | 7,805/13,432 (58%) | 0.019 | 0.63 |
| CCS class | 1 | 8,255/58,278 (14%) | 2,019/13,432 (15%) |  | 1,980/13,432 (15%) | 2,019/13,432 (15%) |  |  |
| CCS class | 2 | 10,507/58,278 (18%) | 2,617/13,432 (19%) |  | 2,570/13,432 (19%) | 2,617/13,432 (19%) |  |  |
| CCS class | 3 | 4,046/58,278 (6.9%) | 852/13,432 (6.3%) |  | 825/13,432 (6.1%) | 852/13,432 (6.3%) |  |  |
| CCS class | 4 | 950/58,278 (1.6%) | 139/13,432 (1.0%) |  | 129/13,432 (1.0%) | 139/13,432 (1.0%) |  |  |
| NYHA class | 1 | 9,342/58,278 (16%) | 2,365/13,432 (18%) | <0.001 | 2,496/13,432 (19%) | 2,365/13,432 (18%) | 0.026 | 0.16 |
| NYHA class | 2 | 23,399/58,278 (40%) | 5,806/13,432 (43%) |  | 5,783/13,432 (43%) | 5,806/13,432 (43%) |  |  |
| NYHA class | 3 | 21,753/58,278 (37%) | 4,753/13,432 (35%) |  | 4,677/13,432 (35%) | 4,753/13,432 (35%) |  |  |
| NYHA class | 4 | 3,784/58,278 (6.5%) | 508/13,432 (3.8%) |  | 476/13,432 (3.5%) | 508/13,432 (3.8%) |  |  |
| Previous PCI | 0 | 56,294/58,278 (97%) | 13,014/13,432 (97%) | 0.30 | 13,020/13,432 (97%) | 13,014/13,432 (97%) | 0.010 | 0.73 |
| Previous PCI | 1 | 91/58,278 (0.2%) | 16/13,432 (0.1%) |  | 20/13,432 (0.1%) | 16/13,432 (0.1%) |  |  |
| Previous PCI | 2 | 68/58,278 (0.1%) | 12/13,432 (<0.1%) |  | 8/13,432 (<0.1%) | 12/13,432 (<0.1%) |  |  |
| Previous PCI | 3 | 1,825/58,278 (3.1%) | 390/13,432 (2.9%) |  | 384/13,432 (2.9%) | 390/13,432 (2.9%) |  |  |
| Poor mobility | 0 | 56,547/58,278 (97%) | 13,054/13,432 (97%) | 0.33 | 13,037/13,432 (97%) | 13,054/13,432 (97%) | 0.008 | 0.53 |
| Poor mobility | 1 | 1,731/58,278 (3.0%) | 378/13,432 (2.8%) |  | 395/13,432 (2.9%) | 378/13,432 (2.8%) |  |  |
| Interval MI | 0 | 57,347/58,278 (98%) | 13,255/13,432 (99%) |  | 13,265/13,432 (99%) | 13,255/13,432 (99%) | 0.009 | 0.86 |
| Interval MI | 1 | 8/58,278 (<0.1%) | 0/13,432 (0%) |  | 0/13,432 (0%) | 0/13,432 (0%) |  |  |
| Interval MI | 2 | 6/58,278 (<0.1%) | 1/13,432 (<0.1%) |  | 1/13,432 (<0.1%) | 1/13,432 (<0.1%) |  |  |
| Interval MI | 3 | 561/58,278 (1.0%) | 104/13,432 (0.8%) |  | 93/13,432 (0.7%) | 104/13,432 (0.8%) |  |  |
| Interval MI | 4 | 356/58,278 (0.6%) | 72/13,432 (0.5%) |  | 73/13,432 (0.5%) | 72/13,432 (0.5%) |  |  |
| Ventilated preoperatively | 0 | 58,113/58,278 (100%) | 13,407/13,432 (100%) | 0.049 | 13,408/13,432 (100%) | 13,407/13,432 (100%) | 0.002 | 0.89 |
| Ventilated preoperatively | 1 | 165/58,278 (0.3%) | 25/13,432 (0.2%) |  | 24/13,432 (0.2%) | 25/13,432 (0.2%) |  |  |
| Cardiogenic shock | 0 | 58,092/58,278 (100%) | 13,411/13,432 (100%) | 0.002 | 13,415/13,432 (100%) | 13,411/13,432 (100%) | 0.008 | 0.52 |
| Cardiogenic shock | 1 | 186/58,278 (0.3%) | 21/13,432 (0.2%) |  | 17/13,432 (0.1%) | 21/13,432 (0.2%) |  |  |
| Inotropes | 0 | 57,961/58,278 (99%) | 13,389/13,432 (100%) | <0.001 | 13,388/13,432 (100%) | 13,389/13,432 (100%) | 0.001 | 0.91 |
| Inotropes | 1 | 317/58,278 (0.5%) | 43/13,432 (0.3%) |  | 44/13,432 (0.3%) | 43/13,432 (0.3%) |  |  |
| Creatinine | Median (Q1, Q3) | 82.00 (82.00, 82.00) | 82.00 (82.00, 82.00) | 0.65 | 82.00 (82.00, 82.00) | 82.00 (82.00, 82.00) | -- | 0.91 |
| EuroSCORE II | Median (Q1, Q3) | 1.17 (0.82, 1.74) | 1.10 (0.78, 1.56) | <0.001 | 1.10 (0.78, 1.62) | 1.10 (0.78, 1.56) | -- | 0.25 |

*PSM: propensity score matching; SMD: standardised mean difference. Continuous variables are median (Q1, Q3); categorical variables are n/N (%). For categorical variables, p values and SMDs are shown on the first level row and compare the full variable distribution. SMD is the post-PSM maximum absolute SMD for multi-level variables. -- indicates not reported in the balance output.*

**Supplementary** Table 2. All isolated MV surgery preoperative characteristics before and after propensity score matching.

| **Characteristic** | **Statistic/level** | **Before PSM** | | | **After PSM** | | | |
| --- | --- | --- | --- | --- | --- | --- | --- | --- |
| **Characteristic** | **Statistic/level** | **Consultant (n=22,725)** | **Trainee (n=2,472)** | **p value** | **Consultant (n=2,472)** | **Trainee (n=2,472)** | **SMD** | **p value** |
| Age, years | Median (Q1, Q3) | 65.40 (56.00, 73.20) | 66.80 (58.10, 73.60) | <0.001 | 66.90 (57.40, 74.50) | 66.80 (58.10, 73.60) | 0.013 | 0.45 |
| Gender | 0 | 12,862/22,725 (57%) | 1,201/2,472 (49%) | <0.001 | 1,214/2,472 (49%) | 1,201/2,472 (49%) | 0.029 | 0.67 |
| Gender | 1 | 9,852/22,725 (43%) | 1,269/2,472 (51%) |  | 1,254/2,472 (51%) | 1,269/2,472 (51%) |  |  |
| Gender | Missing | 11/22,725 (<0.1%) | 2/2,472 (<0.1%) |  | 4/2,472 (0.2%) | 2/2,472 (<0.1%) |  |  |
| BMI | Median (Q1, Q3) | 25.49 (23.04, 28.39) | 25.49 (22.96, 28.46) | 0.34 | 25.49 (23.04, 28.29) | 25.49 (22.96, 28.46) | 0.025 | 0.55 |
| LVEF category | 0 | 4/22,725 (<0.1%) | 0/2,472 (0%) | 0.38 | 0/2,472 (0%) | 0/2,472 (0%) | 0.014 | 0.93 |
| LVEF category | 1 | 4,628/22,725 (20%) | 464/2,472 (19%) |  | 451/2,472 (18%) | 464/2,472 (19%) |  |  |
| LVEF category | 2 | 319/22,725 (1.4%) | 32/2,472 (1.3%) |  | 36/2,472 (1.5%) | 32/2,472 (1.3%) |  |  |
| LVEF category | 3 | 17,201/22,725 (76%) | 1,915/2,472 (77%) |  | 1,924/2,472 (78%) | 1,915/2,472 (77%) |  |  |
| LVEF category | Missing | 573/22,725 (2.5%) | 61/2,472 (2.5%) |  | 61/2,472 (2.5%) | 61/2,472 (2.5%) |  |  |
| Urgency | 1 | 19,476/22,725 (86%) | 2,047/2,472 (83%) | <0.001 | 2,078/2,472 (84%) | 2,047/2,472 (83%) | 0.033 | 0.24 |
| Urgency | 2 | 3,249/22,725 (14%) | 425/2,472 (17%) |  | 394/2,472 (16%) | 425/2,472 (17%) |  |  |
| Diabetes | 0 | 21,354/22,725 (94%) | 2,297/2,472 (93%) | 0.081 | 2,302/2,472 (93%) | 2,297/2,472 (93%) | 0.012 | 0.96 |
| Diabetes | 1 | 399/22,725 (1.8%) | 45/2,472 (1.8%) |  | 42/2,472 (1.7%) | 45/2,472 (1.8%) |  |  |
| Diabetes | 2 | 718/22,725 (3.2%) | 102/2,472 (4.1%) |  | 103/2,472 (4.2%) | 102/2,472 (4.1%) |  |  |
| Diabetes | 3 | 254/22,725 (1.1%) | 28/2,472 (1.1%) |  | 25/2,472 (1.0%) | 28/2,472 (1.1%) |  |  |
| Smoking | 0 | 12,665/22,725 (56%) | 1,340/2,472 (54%) | 0.35 | 1,348/2,472 (55%) | 1,340/2,472 (54%) | 0.019 | 0.80 |
| Smoking | 1 | 8,476/22,725 (37%) | 952/2,472 (39%) |  | 956/2,472 (39%) | 952/2,472 (39%) |  |  |
| Smoking | 2 | 1,584/22,725 (7.0%) | 180/2,472 (7.3%) |  | 168/2,472 (6.8%) | 180/2,472 (7.3%) |  |  |
| Pulmonary disease | 0 | 20,057/22,725 (88%) | 2,133/2,472 (86%) | 0.004 | 2,163/2,472 (88%) | 2,133/2,472 (86%) | 0.035 | 0.21 |
| Pulmonary disease | 1 | 2,668/22,725 (12%) | 339/2,472 (14%) |  | 309/2,472 (13%) | 339/2,472 (14%) |  |  |
| Peripheral vascular disease | 0 | 22,048/22,725 (97%) | 2,404/2,472 (97%) | 0.52 | 2,405/2,472 (97%) | 2,404/2,472 (97%) | 0.003 | 0.93 |
| Peripheral vascular disease | 1 | 677/22,725 (3.0%) | 68/2,472 (2.8%) |  | 67/2,472 (2.7%) | 68/2,472 (2.8%) |  |  |
| Preoperative AF | 0 | 16,148/22,725 (71%) | 1,693/2,472 (68%) | 0.008 | 1,703/2,472 (69%) | 1,693/2,472 (68%) | 0.009 | 0.76 |
| Preoperative AF | 1 | 6,577/22,725 (29%) | 779/2,472 (32%) |  | 769/2,472 (31%) | 779/2,472 (32%) |  |  |
| Neurological dysfunction | 0 | 22,146/22,725 (97%) | 2,401/2,472 (97%) | 0.33 | 2,402/2,472 (97%) | 2,401/2,472 (97%) | 0.002 | 0.93 |
| Neurological dysfunction | 1 | 579/22,725 (2.5%) | 71/2,472 (2.9%) |  | 70/2,472 (2.8%) | 71/2,472 (2.9%) |  |  |
| Previous MI | 0 | 21,921/22,725 (96%) | 2,401/2,472 (97%) | 0.22 | 2,402/2,472 (97%) | 2,401/2,472 (97%) | 0.036 | 0.30 |
| Previous MI | 1 | 741/22,725 (3.3%) | 66/2,472 (2.7%) |  | 69/2,472 (2.8%) | 66/2,472 (2.7%) |  |  |
| Previous MI | 2 | 63/22,725 (0.3%) | 5/2,472 (0.2%) |  | 1/2,472 (<0.1%) | 5/2,472 (0.2%) |  |  |
| Creatinine clearance category | 0 | 1,062/22,725 (4.7%) | 119/2,472 (4.8%) | 0.84 | 121/2,472 (4.9%) | 119/2,472 (4.8%) | 0.019 | 0.92 |
| Creatinine clearance category | 1 | 3,921/22,725 (17%) | 412/2,472 (17%) |  | 395/2,472 (16%) | 412/2,472 (17%) |  |  |
| Creatinine clearance category | 2 | 17,655/22,725 (78%) | 1,933/2,472 (78%) |  | 1,947/2,472 (79%) | 1,933/2,472 (78%) |  |  |
| Creatinine clearance category | 3 | 87/22,725 (0.4%) | 8/2,472 (0.3%) |  | 9/2,472 (0.4%) | 8/2,472 (0.3%) |  |  |
| CCS class | 0 | 18,098/22,725 (80%) | 1,785/2,472 (72%) | <0.001 | 1,764/2,472 (71%) | 1,785/2,472 (72%) | 0.032 | 0.59 |
| CCS class | 1 | 2,604/22,725 (11%) | 388/2,472 (16%) |  | 377/2,472 (15%) | 388/2,472 (16%) |  |  |
| CCS class | 2 | 1,413/22,725 (6.2%) | 216/2,472 (8.7%) |  | 238/2,472 (9.6%) | 216/2,472 (8.7%) |  |  |
| CCS class | 3 | 472/22,725 (2.1%) | 69/2,472 (2.8%) |  | 82/2,472 (3.3%) | 69/2,472 (2.8%) |  |  |
| CCS class | 4 | 138/22,725 (0.6%) | 14/2,472 (0.6%) |  | 11/2,472 (0.4%) | 14/2,472 (0.6%) |  |  |
| NYHA class | 1 | 3,882/22,725 (17%) | 402/2,472 (16%) | <0.001 | 435/2,472 (18%) | 402/2,472 (16%) | 0.036 | 0.51 |
| NYHA class | 2 | 8,314/22,725 (37%) | 879/2,472 (36%) |  | 888/2,472 (36%) | 879/2,472 (36%) |  |  |
| NYHA class | 3 | 8,745/22,725 (38%) | 1,072/2,472 (43%) |  | 1,041/2,472 (42%) | 1,072/2,472 (43%) |  |  |
| NYHA class | 4 | 1,784/22,725 (7.9%) | 119/2,472 (4.8%) |  | 108/2,472 (4.4%) | 119/2,472 (4.8%) |  |  |
| Previous PCI | 0 | 22,097/22,725 (97%) | 2,414/2,472 (98%) | 0.30 | 2,421/2,472 (98%) | 2,414/2,472 (98%) | 0.060 | 0.35 |
| Previous PCI | 1 | 29/22,725 (0.1%) | 1/2,472 (<0.1%) |  | 2/2,472 (<0.1%) | 1/2,472 (<0.1%) |  |  |
| Previous PCI | 2 | 39/22,725 (0.2%) | 1/2,472 (<0.1%) |  | 4/2,472 (0.2%) | 1/2,472 (<0.1%) |  |  |
| Previous PCI | 3 | 560/22,725 (2.5%) | 56/2,472 (2.3%) |  | 45/2,472 (1.8%) | 56/2,472 (2.3%) |  |  |
| Poor mobility | 0 | 22,081/22,725 (97%) | 2,394/2,472 (97%) | 0.36 | 2,395/2,472 (97%) | 2,394/2,472 (97%) | 0.002 | 0.93 |
| Poor mobility | 1 | 644/22,725 (2.8%) | 78/2,472 (3.2%) |  | 77/2,472 (3.1%) | 78/2,472 (3.2%) |  |  |
| Interval MI | 0 | 22,542/22,725 (99%) | 2,461/2,472 (100%) | 0.42 | 2,461/2,472 (100%) | 2,461/2,472 (100%) | 0.008 | >0.99 |
| Interval MI | 1 | 2/22,725 (<0.1%) | 0/2,472 (0%) |  | 0/2,472 (0%) | 0/2,472 (0%) |  |  |
| Interval MI | 2 | 5/22,725 (<0.1%) | 0/2,472 (0%) |  | 0/2,472 (0%) | 0/2,472 (0%) |  |  |
| Interval MI | 3 | 96/22,725 (0.4%) | 5/2,472 (0.2%) |  | 4/2,472 (0.2%) | 5/2,472 (0.2%) |  |  |
| Interval MI | 4 | 80/22,725 (0.4%) | 6/2,472 (0.2%) |  | 7/2,472 (0.3%) | 6/2,472 (0.2%) |  |  |
| Ventilated preoperatively | 0 | 22,597/22,725 (99%) | 2,461/2,472 (100%) | 0.45 | 2,463/2,472 (100%) | 2,461/2,472 (100%) | 0.012 | 0.65 |
| Ventilated preoperatively | 1 | 128/22,725 (0.6%) | 11/2,472 (0.4%) |  | 9/2,472 (0.4%) | 11/2,472 (0.4%) |  |  |
| Cardiogenic shock | 0 | 22,626/22,725 (100%) | 2,460/2,472 (100%) | 0.72 | 2,460/2,472 (100%) | 2,460/2,472 (100%) | 0.000 | >0.99 |
| Cardiogenic shock | 1 | 99/22,725 (0.4%) | 12/2,472 (0.5%) |  | 12/2,472 (0.5%) | 12/2,472 (0.5%) |  |  |
| Inotropes | 0 | 22,540/22,725 (99%) | 2,458/2,472 (99%) | 0.19 | 2,462/2,472 (100%) | 2,458/2,472 (99%) | 0.022 | 0.41 |
| Inotropes | 1 | 185/22,725 (0.8%) | 14/2,472 (0.6%) |  | 10/2,472 (0.4%) | 14/2,472 (0.6%) |  |  |
| Creatinine | Median (Q1, Q3) | 82.00 (82.00, 82.00) | 82.00 (82.00, 82.00) | 0.66 | 82.00 (82.00, 82.00) | 82.00 (82.00, 82.00) | -- | 0.30 |
| EuroSCORE II | Median (Q1, Q3) | 1.10 (0.76, 1.63) | 1.14 (0.83, 1.64) | <0.001 | 1.13 (0.81, 1.70) | 1.14 (0.83, 1.64) | -- | 0.94 |

*PSM: propensity score matching; SMD: standardised mean difference. Continuous variables are median (Q1, Q3); categorical variables are n/N (%). For categorical variables, p values and SMDs are shown on the first level row and compare the full variable distribution. SMD is the post-PSM maximum absolute SMD for multi-level variables. -- indicates not reported in the balance output.*

**Supplementary** Table 3. Isolated MV replacement operative and early postoperative outcomes before PSM, after PSM, and after IPTW.

| **Outcome** | **Before PSM** | **Before PSM** | **Before PSM** | **After PSM** | **After PSM** | **After PSM** | **After IPTW** | **After IPTW** | **After IPTW** |
| --- | --- | --- | --- | --- | --- | --- | --- | --- | --- |
| **Outcome** | **Consultant (n=8,667)** | **Trainee (n=1,226)** | **p** | **Consultant (n=1,223)** | **Trainee (n=1,223)** | **p** | **Consultant (weight=8,667)** | **Trainee (weight=1,235)** | **p** |
| CPB, min | 94 (75, 118) | 96 (82, 114) | 0.006 | 91 (73, 114.5) | 96 (82, 114) | <0.001 | 93 (75, 118) | 96 (82, 115) | <0.001 |
| Cross-clamp, min | 68 (54, 88) | 73 (61, 87) | <0.001 | 66.5 (53, 85) | 73 (61, 87) | <0.001 | 68 (54, 88) | 73 (62, 87) | <0.001 |
| Total length of stay, days | 11 (8, 16) | 10 (8, 16) | 0.40 | 11 (8, 16) | 10 (8, 16) | 0.11 | 11 (8, 16) | 10.04 (8, 16) | 0.48 |
| Mortality | 310/8,616 (3.6%) | 20/1,211 (1.7%) | <0.001 | 58/1,212 (4.8%) | 20/1,208 (1.7%) | <0.001 | 309/8,616 (3.6%) | 33/1,221 (2.7%) | 0.46 |
| Return to theatre | 564/7,728 (7.3%) | 63/1,091 (5.8%) | 0.067 | 88/1,079 (8.2%) | 63/1,089 (5.8%) | 0.030 | 564/7,729 (7.3%) | 68/1,101 (6.2%) | 0.21 |
| Bleeding/tamponade return to theatre | 419/8,667 (4.8%) | 49/1,226 (4.0%) | 0.20 | 65/1,223 (5.3%) | 49/1,223 (4.0%) | 0.12 | 420/8,667 (4.8%) | 53/1,235 (4.3%) | 0.41 |
| Postoperative CVA/TIA | 160/7,628 (2.1%) | 25/1,123 (2.2%) | 0.51 | 23/1,058 (2.2%) | 25/1,120 (2.2%) | 0.52 | 160/7,628 (2.1%) | 25/1,136 (2.2%) | 0.41 |
| Postoperative dialysis | 264/7,629 (3.5%) | 20/1,049 (1.9%) | 0.008 | 39/1,067 (3.7%) | 20/1,047 (1.9%) | 0.015 | 263/7,627 (3.4%) | 22/1,059 (2.1%) | 0.057 |
| Deep sternal wound infection | 15/2,241 (0.7%) | 1/222 (0.5%) | >0.99 | 1/289 (0.3%) | 1/221 (0.5%) | >0.99 | 15/2,222 (0.7%) | 2/238 (0.6%) | 0.96 |
| DSWI treatment | 11/2,237 (0.5%) | 1/222 (0.5%) | 0.46 | 1/289 (0.3%) | 1/221 (0.5%) | 0.68 | 11/2,218 (0.5%) | 2/238 (0.6%) | 0.31 |

Consultant: consultant first operator; trainee: trainee first operator; PSM: propensity score matching; IPTW: inverse probability of treatment weighting; weight: IPTW weighted total. Continuous variables are median (Q1, Q3); categorical variables are n/N (%).

**Supplementary** table 4. Isolated MV repair operative and early postoperative outcomes before PSM, after PSM, and after IPTW.

| **Outcome** | **Before PSM** | **Before PSM** | **Before PSM** | **After PSM** | **After PSM** | **After PSM** | **After IPTW** | **After IPTW** | **After IPTW** |
| --- | --- | --- | --- | --- | --- | --- | --- | --- | --- |
| **Outcome** | **Consultant (n=14,046)** | **Trainee (n=1,246)** | **p** | **Consultant (n=1,243)** | **Trainee (n=1,243)** | **p** | **Consultant (weight=14,046)** | **Trainee (weight=1,251)** | **p** |
| CPB, min | 98 (78, 126) | 98 (81, 120) | 0.48 | 98 (78, 126) | 98 (81, 120) | 0.75 | 98 (78, 126) | 98 (80, 120) | 0.77 |
| Cross-clamp, min | 74 (58, 95) | 74 (60, 91) | 0.97 | 73 (57, 95) | 74 (60, 91) | 0.58 | 74 (58, 95) | 75 (60, 92) | 0.40 |
| Total length of stay, days | 8 (7, 12) | 9 (7, 12) | 0.26 | 9 (7, 12) | 9 (7, 12) | 0.20 | 8.04 (7, 12) | 8 (7, 11) | 0.40 |
| Mortality | 159/13,941 (1.1%) | 9/1,234 (0.7%) | 0.19 | 20/1,232 (1.6%) | 9/1,231 (0.7%) | 0.040 | 160/13,941 (1.1%) | 8/1,239 (0.6%) | 0.081 |
| Return to theatre | 629/12,851 (4.9%) | 49/1,063 (4.6%) | 0.68 | 58/1,124 (5.2%) | 49/1,060 (4.6%) | 0.56 | 630/12,845 (4.9%) | 43/1,082 (4.0%) | 0.18 |
| Bleeding/tamponade return to theatre | 459/14,046 (3.3%) | 39/1,246 (3.1%) | 0.79 | 46/1,243 (3.7%) | 39/1,243 (3.1%) | 0.44 | 460/14,046 (3.3%) | 34/1,251 (2.7%) | 0.26 |
| Postoperative CVA/TIA | 139/12,139 (1.1%) | 15/1,108 (1.4%) | 0.34 | 15/1,056 (1.4%) | 15/1,105 (1.4%) | 0.76 | 140/12,136 (1.2%) | 15/1,108 (1.4%) | 0.35 |
| Postoperative dialysis | 184/12,307 (1.5%) | 10/1,019 (1.0%) | 0.19 | 19/1,064 (1.8%) | 10/1,016 (1.0%) | 0.12 | 185/12,304 (1.5%) | 10/1,036 (1.0%) | 0.20 |
| Deep sternal wound infection | 17/4,826 (0.4%) | 0/281 (0%) | >0.99 | 3/420 (0.7%) | 0/281 (0%) | 0.28 | 17/4,824 (0.4%) | 0/302 (0%) | 0.30 |
| DSWI treatment | 15/4,824 (0.3%) | 0/281 (0%) | >0.99 | 3/420 (0.7%) | 0/281 (0%) | 0.71 | 15/4,822 (0.3%) | 0/302 (0%) | 0.63 |

Consultant: consultant first operator; trainee: trainee first operator; PSM: propensity score matching; IPTW: inverse probability of treatment weighting; weight: IPTW weighted total. Continuous variables are median (Q1, Q3); categorical variables are n/N (%).
